# The Impact of a Range‐Shifting Predator Is Affected by Prey Preference and Composition

**DOI:** 10.1002/ece3.72538

**Published:** 2025-11-20

**Authors:** Kyle J. Suen, Ryan A. Beshai, Cascade J. B. Sorte

**Affiliations:** ^1^ Ecology & Evolutionary Biology University of California Irvine California USA

**Keywords:** apparent facilitation, community context, impact, *Mexacanthina lugubris*, predation, range shift

## Abstract

Global biodiversity is undergoing a grand reshuffling with species across taxa and biomes shifting their ranges in response to climate change. Research on the ecological impacts of range‐shifting species has prioritized linking the traits of the range‐shifting species themselves to impacts, with studies giving more limited attention to the characteristics of the recipient community and its prey composition. Understanding how community composition and structure can alter the impact of novel species via prey preferences is important for predicting and managing ecological changes. We used the range‐shifting predatory whelk *Mexacanthina lugubris* as a case study to investigate how prey composition might influence prey preferences and overall range shift impacts on prey species. Specifically, we hypothesized that *Mexacanthina lugubris'* consumptive effects on a single prey species would be modified by the presence (or abundance) of a second prey species. We tested this hypothesis via a field experiment in southern California, where we caged whelks at a gradient of densities and observed their predation on mussels, barnacles living on bare rock, and barnacles living on mussels over 8 weeks. We found that *Mexacanthina lugubris* consumed barnacles on bare rock preferentially before consuming barnacles on mussels and mussels themselves. Our findings demonstrate that the presence of mussels (which act as both habitat and prey) can mitigate the overall impact of the range‐shifting predator by altering accessibility of barnacle prey. This context‐dependent attenuation of predator impacts highlights a form of apparent facilitation among prey and underscores the importance of considering recipient community traits when assessing or managing the ecological consequences of range‐shifting species.

## Graphical abstract

Understanding how community composition and structure can alter the impact of novel species via prey preferences is important for predicting and managing ecological changes. We used the range‐shifting predatory whelk *Mexacanthina* lugubris as a case study to investigate how prey composition might influence prey preferences and overall range shift impacts on prey species. We found that *Mexacanthina* lugubris consumed barnacles on bare rock preferentially before consuming barnacles on mussels and mussels themselves, demonstrating the role of community composition in range‐shift impact alteration.

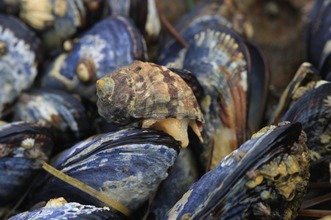

## 1 Introduction

Rising global temperatures are affecting ecosystems worldwide, including by driving shifts in the ranges of species (Lenoir and Svenning 2015; Chen et al. 2011). Temperature is an important factor in defining the areas where a species can exist (Hutchins 1947; Araújo and Pearson 2005). As temperatures increase globally, species are undergoing poleward shifts toward areas that were previously inhospitable due to their lower temperatures (Henry and Sorte 2022). The arrival of a range‐shifting species in a novel community beyond its historic range edge can lead to strong negative impacts on recipient communities (Henry and Sorte 2022). Range‐shifting species have been observed competing with, consuming, and parasitizing native species (Sorte et al. 2010; Nackley et al. 2017). When native ecosystems are affected, economic development, food security, and human health can also be negatively impacted (Pecl et al. 2017). For instance, climate warming is allowing the oyster parasite *Perkinsus marinus* to spread northward of its original range, causing high mortality in oysters and negative socioeconomic effects (Ford and Smolowitz 2007; Henry and Sorte 2022). Thus, understanding when and where impacts may be mitigated can help us best anticipate and respond to changes in climate.

Most studies aimed at predicting impacts of invasive and range‐shifting species only consider the characteristics of the novel species (Lodge 1993; Vermeij 1996; Nyberg and Wallentinus 2005). For instance, high competitive ability has been used as an explanation for patterns of native species reduction by a range‐shifting damselfly (Fitt and Lancaster 2017). Meta‐analyses and reviews have also focused on species‐centric characteristics like competitiveness to assess which non‐native, invasive plants had the highest potential to be ecologically disruptive (Rockwell‐Postel et al. 2020; Butt and Gallagher 2018). In the marine environment, the impact of a range‐shifting crab on oyster populations was predicted based on crab density and metabolic rate (Hollebone and Hay 2007).

Beyond the traits of novel species, characteristics of the recipient community—including prey composition—might also play a role in the impact of novel species. There is a rich literature about the context‐dependency of species interactions, with the magnitude and even the sign of interactions (positive versus negative) depending on abiotic conditions (Chamberlain et al. 2014; Maron et al. 2014; Tonkin et al. 2016; Tomiolo and Ward 2018a) and biotic attributes (Liu and Gaines 2022; MacDougall et al. 2018). It is perhaps not surprising, then, that community context can play an important role in the impact of novel species arriving via range shifts (Tomiolo and Ward 2018b) or human‐mediated introductions (Colautti and MacIsaac 2004; Ruesink 2003; Liao et al. 2015). For instance, researchers monitoring the impact of an introduced clam species (*Ruditapes philippinarum*) in Europe found that while the clam had become an important ecosystem engineer, its role in sediment mixing depended on the densities of native macroalgae and community composition (de Moura Queirós et al. 2011). Novel species may also experience population growth due to release from the competitors and predators in their native ranges (Middleton 2019; Bossdorf 2013; Mitchell et al. 2006; Zeidberg and Robison 2007). This advantage can be experienced by range‐shifting species as well (Berg et al. 2010; Engelkes et al. 2008). Prey composition can also influence the impacts of range‐shifting species. For example, natural resource managers could identify areas that may face high impact by learning the prey preferences of range‐shifting predators and mapping that onto community composition in the region of interest (Green 2025). It is therefore surprising that the role of recipient community traits has been relatively poorly studied for even species invasions, a relatively well‐established field, and even less so for species range shifts (Lany et al. 2017; Kumschick et al. 2015; Ricciardi et al. 2021; Pyšek et al. 2020; Nackley et al. 2017; Wallingford et al. 2020).

**FIGURE 1:**
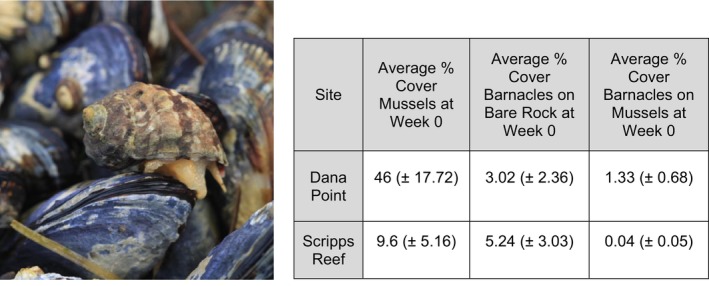
Left: Photograph of *Mexacanthina lugubris* on top of *Mytilus californianus* . Taken by RAB. Right: Average percent cover of prey in plots (mussels [ *Mytilus californianus* ] and acorn barnacles [*Balanus* and *Chthamalus* spp.]). Percent cover of acorn barnacles is further separated by substrate (on bare rock or on mussels).

Here, we explore the role of prey composition and preference in modifying range shift impacts using a predatory snail as a model species. *Mexacanthina lugubris* (hereafter referred to as *Mexacanthina*) is a whelk that lives in rocky shore communities along the North American west coast (Figure 1). *Mexacanthina* has undergone both a rapid and recent range shift: the species has been documented as far north as Santa Monica, California, USA (Beshai et al. in press), indicating more than a 200 km northward increase from its historical range boundary established 50 years ago (Fenberg et al. 2014). *Mexacanthina* has a varied diet, but primarily preys on barnacles due to their specialized physiology: these whelks possess a labral spine that is used to puncture the opercular plates of barnacles. *Mexacanthina*'s physiology also results in relatively lower energy costs when feeding on barnacles (Lively 1986; Jarrett 2008; Jarrett 2009; Fenberg et al. 2014, 2023). Whelks can exert strong impacts on their prey (Navarrete 1996). As a result, *Mexacanthina* may have strong effects on communities in its expanded range of southern California (Beshai et al. in press).

In this study, we tested the hypothesis that *Mexacanthina* will prefer to eat barnacles, but the presence and abundance of mussels will affect whelk predation on barnacles. Likewise, the presence of barnacles may affect feeding patterns on mussels. Mussels, another prey of *Mexacanthina* (Becker 2005), are foundation species that provide habitat on their relatively large shells and in their aggregations of structurally complex “beds” (Borthagaray and Carranza 2007), allowing them to facilitate other species including by protecting associated species from predation (Gutiérrez et al. 2003; Witman 1985; Gosselin and Chia 1995; Borthagaray and Carranza 2007). As barnacles are known to reside on mussels, we predict that barnacles that use the beds as substrate are protected from predation by *Mexacanthina* (Puccinelli and McQuaid 2021). Because mussels are more abundant in *Mexacanthina*'s expanded range in southern California than in *Mexacanthina*'s historical range in Baja California, Mexico (Beshai et al. in press), it is important to understand whether and how *Mexacanthina*'s presence affects prey populations in its expanded range.

## 2 Materials and Methods

To evaluate how the prey composition (i.e., relative abundance of barnacles and mussels) affects the prey preference of range‐shifting whelks, we conducted a caging experiment at two coastal sites in southern California, USA: Dana Point (33.460, −117.715) and Scripps Reef (32.871, −117.253). Past surveys at rocky intertidal sites along the west coast (Beshai et al. in press) had shown that these two sites were ideal for studying interactions between prey species and *Mexacanthina*, as both sites contain relatively high abundances of California mussels ( *Mytilus californianus* ), bare exposed rock, acorn barnacles (*Balanus and Chthamalus* spp.), and *Mexacanthina*. Scripps Reef contains relatively lower abundances of mussels making it representative of a low‐mussel site as compared to Dana Point which was a high‐mussel site (Figure 1).

We established plots at each site to measure the effect of whelk density on native prey populations and to observe potential changes in prey preference. At each field site, five square 0.1 m^2^ plots were established on flat, bare rock surfaces in the location with maximum *Mexacanthina* abundance, at a shore height of 0.76 ± 0.01 m and 1.03 ± 0.11 m at Dana Point and Scripps Reef, respectively. At each of the two field sites, we randomly assigned each plot to one of five treatment levels using a gradient/regression experimental design, which involved adding zero, one, three, six or twelve *Mexacanthina* to the plot.

To ensure that the average whelk size was consistent across treatments, we separated *Mexacanthina* into size classes. We measured along the longest axis for their size, and then grouped individuals into small, medium, and large size classes, based on the size distribution of individuals encountered on the day of cage deployment. Total lengths of whelks in each size classification are listed in Appendix 1 (Table A1). We placed equal numbers of each size class in each whelk‐addition cage, except for the one‐whelk cage where we placed one medium individual. The mussels and barnacles that were within the cages represented naturally occurring size distributions at each respective site (Figure 1).

Square steel mesh cages were then installed on each plot, and gaps in the cages were filled in with rocks and epoxy resin to prevent the entry or escape of whelks during the experiment. Open and partial cage treatments were also included to test for any effect of the cage on whelk predation or shellfish mortality. Because no caging effects were detected (Beshai et al. in press), these control treatments are not considered in the current study. Every 2 weeks, the cage was removed, and the plots were surveyed in two ways. First, photographs were taken to estimate barnacle mortality, as described below. Second, mussel mortality was estimated visually in the field. Mussel mortality due to whelk predation was determined based on observation of a distinct hole in the shell, which is created by *Mexacanthina* when drilling and consuming mussels. Dead mussels were collected and removed from the cages after recording. If a plot was missing any *Mexacanthina* individuals, a new, medium‐sized *Mexacanthina* was located and placed in the plot; if an extra individual was found then a medium‐sized *Mexacanthina* would be taken out. On two and one occasions, one *Mexacanthina* needed to be added or removed, respectively, and in one instance, three whelks needed to be added (to the Dana Point twelve‐whelk cage during the second week). Cages were then re‐installed. Plot maintenance and monitoring occurred every 2 weeks for a total of 8 weeks, from May to July 2023.

The consumption of *Mexacanthina* on barnacle populations in each plot was measured from observations of photos taken at both sites. A total of 48 top‐down photos were taken across all plots and the two sites (two sites × five plots × five monitoring events, with two plots missing from our dataset: the “three whelk” cage at Dana Point during the second week of observations, and the “twelve whelk” cage at Scripps Reef during the day of deployment. The “twelve whelk” cage at Scripps Reef was omitted from the analysis of barnacle consumption due to the missing photograph, but was included for mussel consumption analysis). Using the image analysis software ImageJ, we classified the pixel area of acorn barnacles in each of the photos into four groups: (1) live acorn barnacles on bare rock, (2) live acorn barnacles on mussels, (3) dead acorn barnacles on bare rock, and (4) dead acorn barnacles on mussels.

The relationship between predator density and changes in prey abundance were assessed using linear regression analyses run in MATLAB (23.2.0.2485118 (R2023b) Update 6). We assessed the mortality of barnacles (by habitat type: bare rock or mussels as substrate) and mussels as prey by evaluating the change in percentage of live barnacles or change in number of dead mussels as a function of *Mexacanthina* density, which allowed us to attribute mortality to predation by *Mexacanthina*. The percentage of live barnacles was defined as the ImageJ‐generated photo pixel area of live acorn barnacles on a specific substrate (bare space or mussels) divided by the total photo pixel area of acorn barnacles on that substrate (live + dead) multiplied by 100. Barnacles that could not be characterized as living or dead were not included in this analysis. The number of dead mussels in each plot was an absolute (not proportional) value. *Mexacanthina* density was defined as the moving average of *Mexacanthina* recorded over every observation for a specific cage treatment (rather than the target treatment values, as *Mexacanthina* would sometimes escape the cage as described above). These three analyses (for live barnacles on rock, live barnacles on mussels as substrate, and dead mussels) were performed after calculating the change over 2, 4, 6, and 8 weeks. All analyses were performed separately for each site, which we determined was appropriate given different community contexts that could have influenced the overall predator impacts and rate of those impacts (such as habitat heterogeneity, prey species abundance, and prey dispersion patterns).

We also evaluated whether *Mexacanthina* preyed selectively by calculating a selectivity index. Selective predation occurs when the relative frequency of a prey type in a diet is different from the relative frequency of the prey type in the environment (Chesson 1978). Because it functions well when there are differences in relative abundance of food types (Lechowicz 1982), Chesson's alpha (*α*) was chosen for the selectivity index:

$$\alpha_{i}=\frac{r_{i}/p_{i}}{\sum_{i}r_{i}/p_{i}}$$

where *i* is the specific prey type (Chesson 1978). Because we were comparing two prey items (barnacles on bare rock vs. barnacles on mussels as substrate), we performed one calculation of selectivity for barnacles on bare rock. We used the proportion of available barnacle prey on bare rock (*p*_bare rock_), the proportion of consumed barnacles on bare rock (*r*_bare rock_), the proportion of available barnacle prey on mussels (*p*_mussel_), and the proportion of consumed barnacles on mussels (*r*_mussel_). The proportion of available barnacle prey on a substrate was defined as the photo pixel area of live acorn barnacles on the respective substrate divided by the total photo pixel area of live barnacles in the cage. Live barnacles in the cage included barnacles living on both bare rock and on mussels. The proportion of consumed barnacles was defined as the change in photo pixel area of dead barnacles on the respective substrate over a time range divided by the total change in photo pixel area of dead barnacles in the cage over the same time range. Likewise, dead barnacles in the cage included barnacles on both bare rock and on mussels. Because the change in percent cover of dead barnacles denoted the amount of eaten barnacles, this value was bounded at zero such that negative values were treated as zero‐values. The change in the selectivity index of barnacles on bare rock was then evaluated as a function of *Mexacanthina* density. Selectivity indices that fall above 1/*n* indicate preference for barnacles on bare rock by the *Mexacanthina* in the cage, whereas indices that fall below 1/*n* denote, in this case, preference for barnacles on mussels as substrate, with *n* being the number of types of food in the sample (Chesson 1978). We note that when the zero‐whelk cage is not included in the preference regression analysis, there is no change in our conclusions.

**FIGURE A2:**
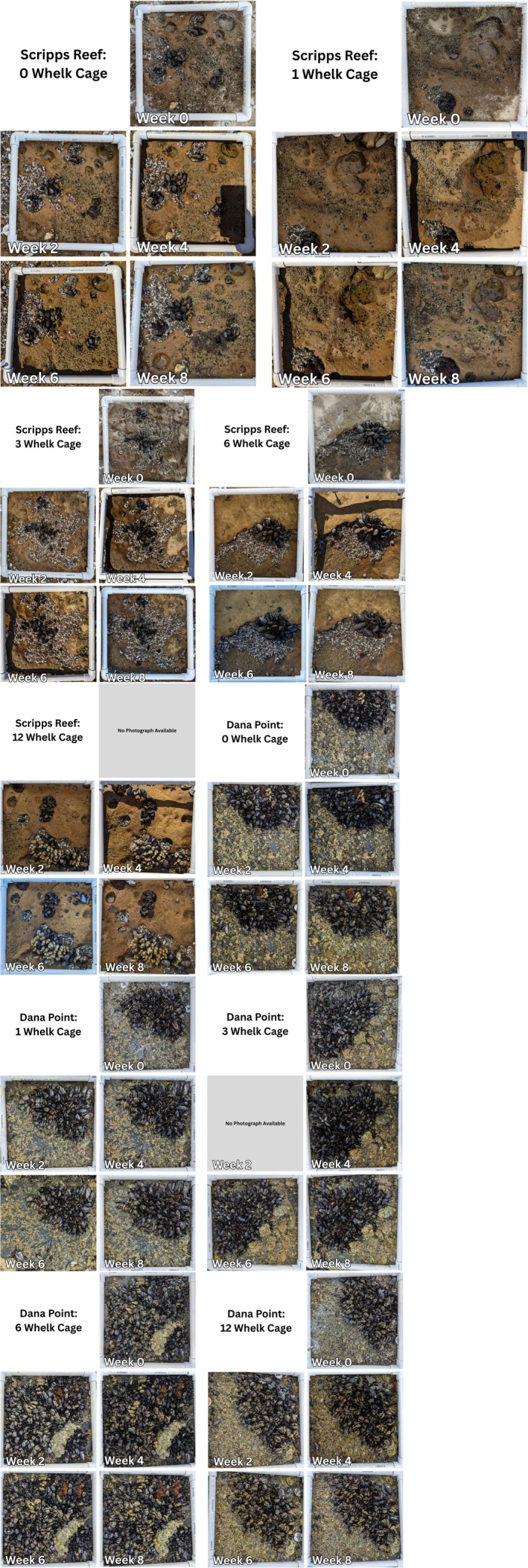
Photographs of Each Plot. Photographs were taken at the beginning of observation, and after whelks in cages had been removed. *n* = 48 photos (two sites × five plots × five monitoring events, with two plots missing from our dataset: The “three whelk” cage at Dana Point during the second week of observations, and the “twelve whelk” cage at Scripps Reef during the day of deployment).

For all photographs, there were barnacles on mussel beds that we were unable to classify as living or dead because they resided on the sides of mussels and therefore could not be seen in the photographs. All barnacles on bare rock were classified due to the rock faces on all plots being visible in the photograph view. A follow‐up survey was conducted to determine whether our inability to classify barnacles on the sides of mussels would be likely to influence the conclusions of the study (See Appendix 2). All photographs of plots over time are displayed in Appendix 2 Figure A2.

## 3 Results

**FIGURE 2:**
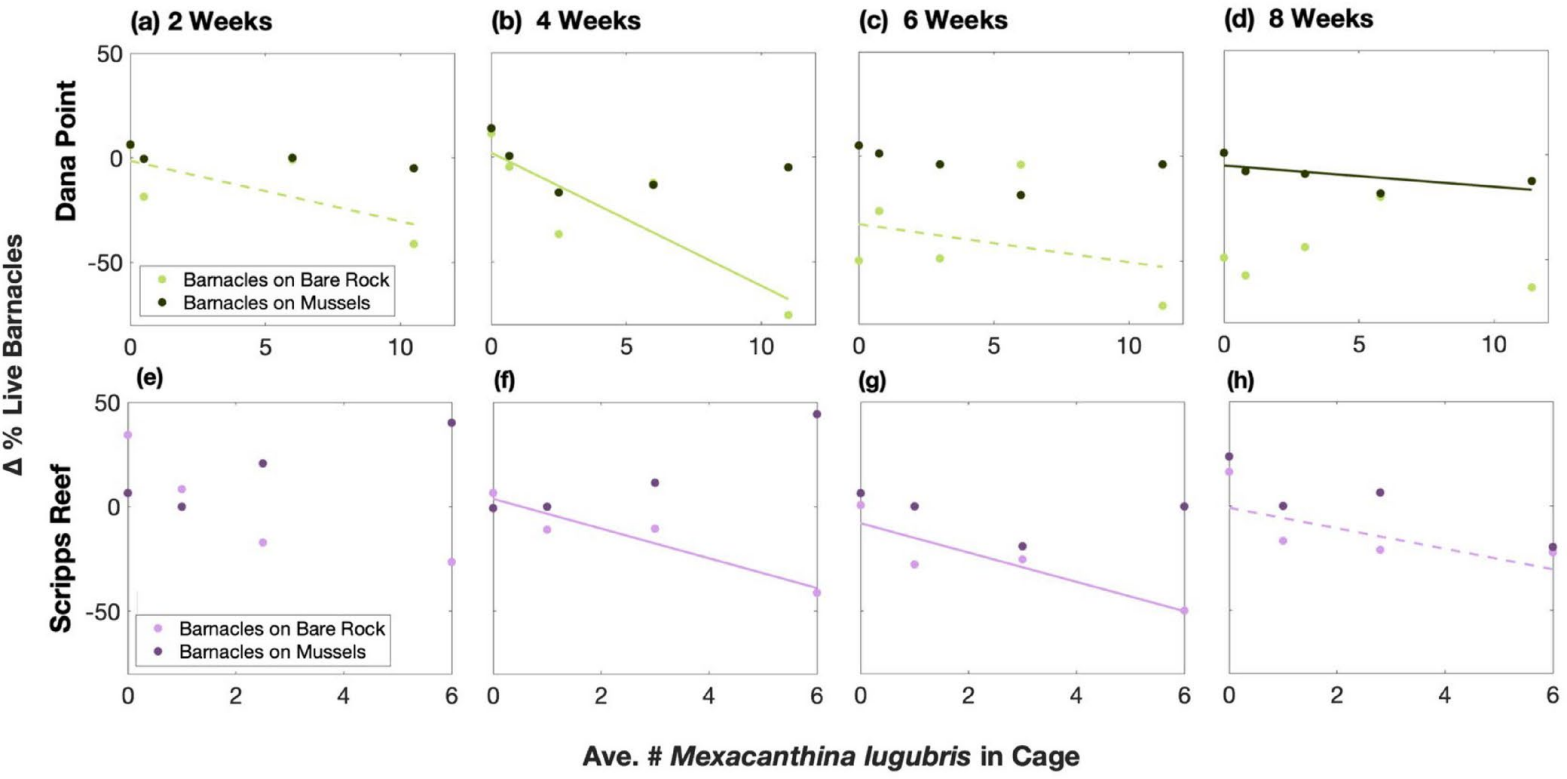
Changes in the percent cover of live barnacles as a function of the average number of *Mexacanthina lugubris* in cages over (a, e) two, (b, f) four, (c, g) six, and (d, h) 8 weeks. Data points indicate the change in live barnacles in a plot from the start of the experiment (time 0) on either bare rock (light green at Dana Point, light purple at Scripps Reef) or mussels (dark green at Dana Point, dark purple at Scripps Reef), with vertically aligned points coming from the same plot. Note that the maximum level of *Mexacanthina* abundance differs between sites (*n* = 5 plots at Dana Point except [a], which was missing a 3‐whelk cage photo; *n* = 4 plots at Scripps Reef). Significant trends are indicated by solid lines and near‐significant trends (0.05 < *p* < 0.1) are indicated by a dotted line.

**FIGURE 3:**
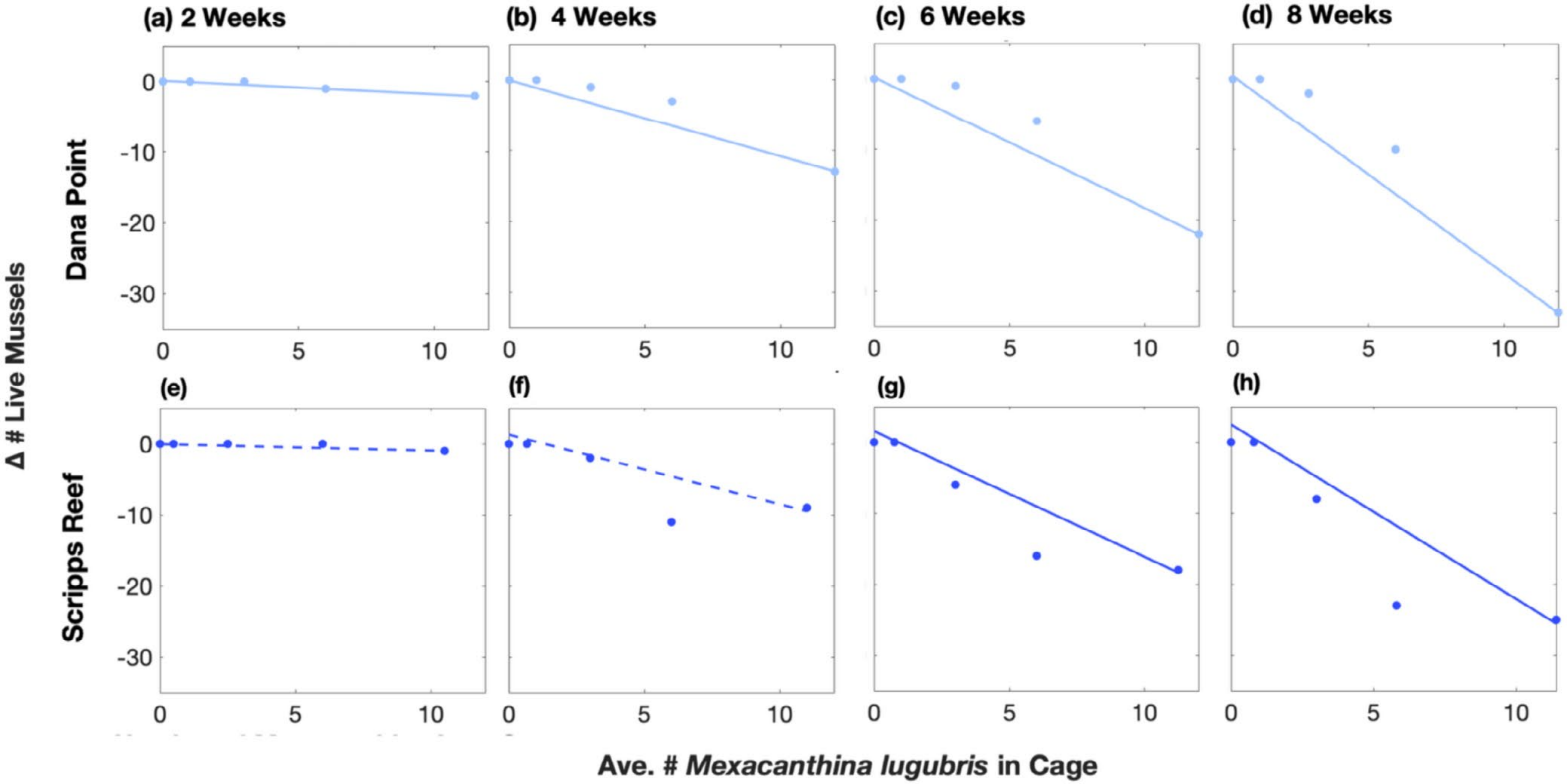
Changes in the number of live mussels via consumption as a function of the average number of *Mexacanthina lugubris* in cages over (a, e) two, (b, f) four, (c, g) six, and (d, h) 8 weeks. Data points indicate the number of dead mussels found in plots (represented as a negative value) since the start of the experiment (time 0) at either Dana Point (light blue) or Scripps Reef (dark blue), with vertically aligned points coming from the same plot (*n* = 5 plots). Significant trends are indicated by solid lines and near‐significant trends (0.05 < *p* < 0.1) are indicated by a dotted line.

*Mexacanthina* consumed barnacles on bare rock preferentially before consuming barnacles on mussels and mussels themselves. In the first 4 weeks of the experiment, there was a high level of mortality of barnacles living on bare rock; at Dana Point, almost 80% of these barnacles in the highest‐density cage died (Figure 2b). Importantly, barnacle mortality on bare rock was significantly related to *Mexacanthina* density at this point in the experiment (Dana Point *p* = 0.0094; Scripps Reef *p* = 0.0089; Figure 2b,f), indicating that mortality was due to consumption. Over these 4 weeks, *Mexacanthina* predation on barnacles on mussels and mussels themselves was minimal, especially in the first 2 weeks (Figure 3a,e). However, between 4 and 8 weeks, barnacles on bare rock experienced little additional mortality (Figure 2c,d,g,h). In contrast, consumption of barnacles living on mussels increased with *Mexacanthina* density, particularly at Dana Point (*p* = 0.032; Figure 2d). Mussels in higher‐density cages were depleted at a much faster rate than they were in low‐density cages, which led to large variation between the different cage treatments. At 8 weeks, mussel mortality was also correlated with *Mexacanthina* density (Dana Point *p* = 0.025; Scripps Reef *p* = 0.004; Figure 3d,h).

**FIGURE 4:**
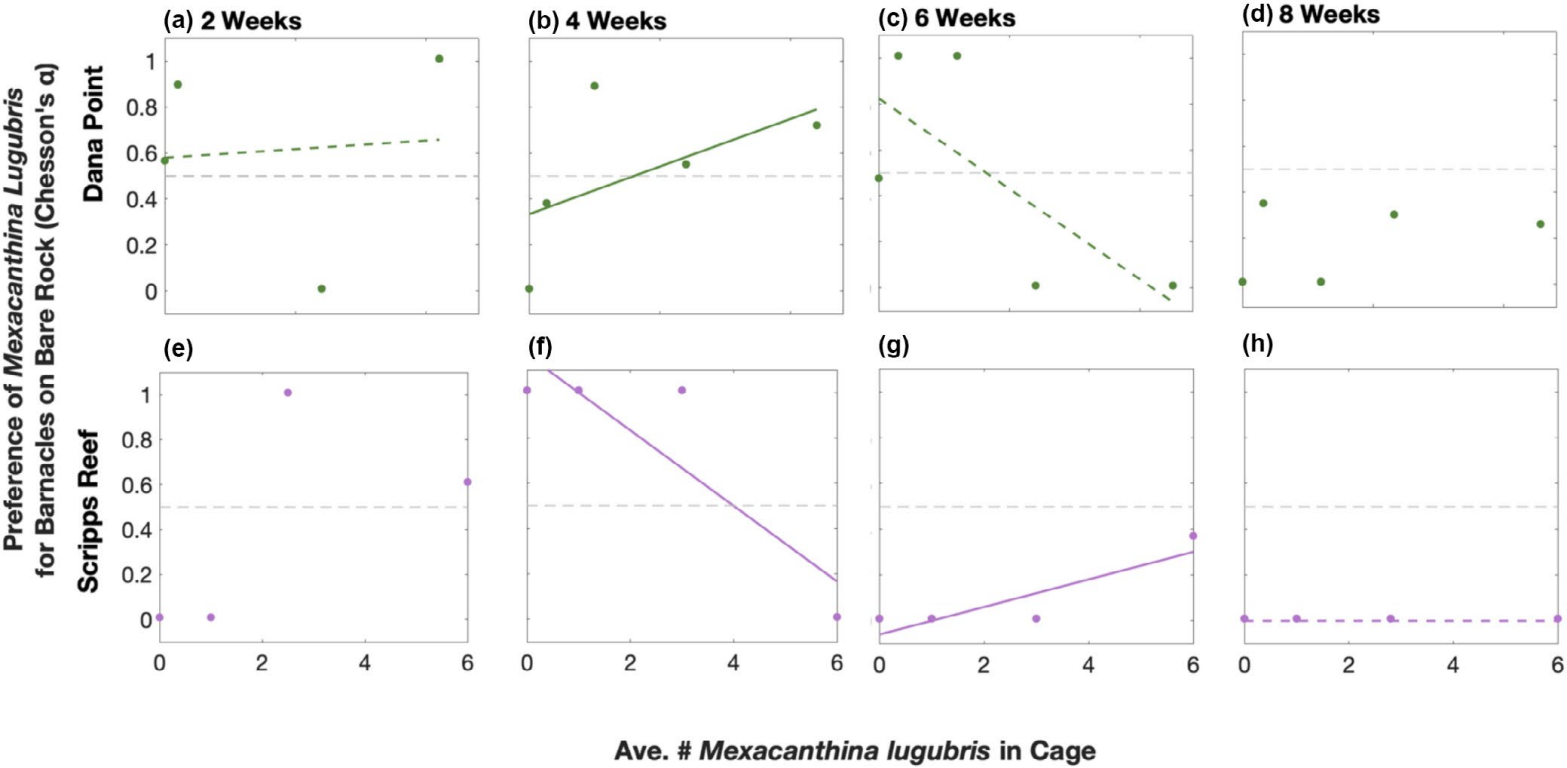
Changes in *Mexacanthina lugubris* preference for barnacles on bare rock compared to barnacles on mussels as a function of the average number of *Mexacanthina lugubris* in cages over (a, e) two, (b, f) four, (c, g) six, and (d, h) 8 weeks. Data points represent the value of the selectivity index (Chesson's alpha), with points above the gray dotted line indicating a preference for barnacles on bare rock based on consumption since the start of the experiment (time 0); data points below the gray dotted line indicate a preference for barnacles on mussels. Note that the maximum level of *Mexacanthina* abundance differs between sites (*n* = 5 plots at Dana Point except [a], which was missing a 3‐whelk cage photo; *n* = 4 plots at Scripps Reef). Significant trends are indicated by solid lines and near‐significant trends (0.05 < *p* < 0.1) are indicated by a dotted line.

Preference for barnacles on bare rock as a function of prey availability also followed a similar pattern as total consumption of barnacles on bare rock. At the beginning of the experiment, *Mexacanthina* exhibited a preference for barnacles on bare rock over barnacles on mussels in all cages (Figure 4a,b,e,f). Starting in the higher *Mexacanthina* density cages, preference then shifted away from barnacles on bare rock as *Mexacanthina* began consuming barnacles living on mussels (Figure 4c,f). By the end of the experiment, there was no preference for barnacles on bare rock in any of the cages (starting at Week Six in Scripps Reef and at Week Eight at Dana Point; Figure 4d,g).

## 4 Discussion

This experiment demonstrated that prey composition influenced the feeding preference of a range‐shifting species; specifically, under the conditions tested, we showed that the consumption of shellfish prey depended on the co‐occurring prey species, with mussel prey escaping predation potentially indefinitely if barnacle abundance is sufficiently high to meet predator demand. We found that *Mexacanthina* prefer to eat acorn barnacles on bare rock over barnacles on mussels. As a result, *Mexacanthina* consume acorn barnacles on bare rock before moving to acorn barnacles on mussels and the mussels themselves. This supports past studies that show that *Mexacanthina* specialize in consuming barnacles (Lively 1986; Jarrett 2008, 2009; Fenberg et al. 2014; Fenberg et al. 2023). Predators such as whelks might have difficulty accessing and moving within dense mussel beds compared to flat, bare rock, suggesting that whelks may prefer prey that is on bare rock more than prey in or on mussel beds (Stephen Gosnell et al. 2012). Acorn barnacles have higher survivorship in the presence of mussel beds, which protect them from thermal stress (Stephens and Bertness 1991), and growth has been found to be higher for a barnacle species when it resided on live mussels (Buschbaum 2001).

Shifts in prey type preference and consumption occurred at different time points. Notably, barnacles on bare rock experienced whelk density‐dependent predation longer at Scripps Reef than at Dana Point, with no relationship between whelk density and consumption of barnacles on mussels appearing at Scripps Reef during the experiment. Plots at Scripps Reef contained larger areas of exposed bare rock and fewer mussels (Figure 1), which could explain the longer‐lasting density‐dependent consumption of barnacles on bare rock. Put another way, barnacles on bare rock may have been a preferred prey for longer because they were more available than barnacles on mussels. Predation of barnacles on bare rock might also be more time‐consuming than eating barnacles on mussels if whelks preying on bare rock are more exposed to thermal stress from lack of mussel shelter and because prey are more dispersed (Stephens and Bertness 1991).

*Mexacanthina* may prefer prey on bare rock over mussels due to differences in energy expenditure. Mussels may provide refuge for barnacles by being a less optimal food source to find and consume, since food that has the highest energy gain and requires the lowest energy cost tends to be consumed first (Hughes 1980; Pyke 2010). To consume mussels, *Mexacanthina* must drill through thick shells, which requires a longer handling time than barnacles and therefore more energy. This also increases the chance of interference from competitors (Hughes, and de B. Dunkin, S. 1984). When compared to mussels, barnacles as prey support higher shell growth rates despite their relatively lower energy supply (Burrows and Hughes 1991). *Mexacanthina*'s specialized physiology for eating barnacles also results in relatively lower energy costs (Lively 1986; Jarrett 2008; Jarrett 2009; Fenberg et al. 2014; Fenberg et al. 2023). Studies have shown that the dogwhelk ( *Nucella lapillus* ), a closely related whelk that attacks similar prey as *Mexacanthina*, chooses barnacles over mussels to maximize energy (Dernbach and Freeman 2015). This pattern of prey selectivity and predation refuge might, therefore, be conserved across predatory whelks as their ranges shift (Flagor and Bourdeau 2018; Zacherl et al. 2003; Rivadeneira and Fernández 2005) as for other predators who encounter prey with the potential for facilitative feedbacks (Iwashita et al. 2022; Cuthbert et al. 2018; Castorani and Hovel 2015).

In this study, we used image analysis to estimate the proportion of live barnacles in each cage as an imperfect yet effective indicator of *Mexacanthina* consumption rate. To ensure that barnacle mortality rates could be attributed to consumption by *Mexacanthina*, cages were placed close to each other and at the tide height of maximum whelk abundance, with the treatment being *Mexacanthina* abundance. Barnacle mortality was correlated with the abundance of *Mexacanthina*, indicating that *Mexacanthina* consumption was responsible for barnacle mortality. However, we note that there was a small amount of unattributed mortality in the cage treatment with zero *Mexacanthina*, particularly at Dana Point, which may have been due to an incursion of juvenile whelks that were small enough to enter the cage. It is also important to note that a comparison between data gathered from observations of photos vs. in the field showed that these methods yielded statistically equivalent results (Appendix 2; Table A2).

Although *Mexacanthina* exhibits preferences for easy‐to‐access barnacles, based on the predation rates observed in this experiment and documented barnacle growth rates (Pineda 1994; Sanford and Menge 2001), it seems unlikely that *Mexacanthina* will drive dramatic declines in the barnacle population in southern California. In southern California, current *Mexacanthina* density averages ~3 individuals per m^2^ in *Mexacanthina*'s areas of expansion (Beshai et al. in press; Wallingford and Sorte 2022). Prey levels may be sustainable at this whelk density. Based on the results of this study and average *Mexacanthina* densities in southern California (which is a fraction of that in the one‐whelk treatment), the population of barnacles on bare rock would theoretically decrease by < 10% at both studied sites. This is unlikely to overwhelm the barnacle population given that acorn barnacles, such as *Chthamalus dalli* , recruit throughout the year (Pineda 1994) and recruits can grow as much as 95% larger over the course of 8 weeks (Sanford and Menge 2001). However, peak *Mexacanthina* densities (128 individuals per m^2^) would approximate the density of the twelve‐whelk cage, leading to up to 45% of barnacles on bare rock being consumed. While barnacles on mussels experienced relatively lower consumption rates in this experiment, the recruitment and growth rates of barnacles do not appear to change with substrate: barnacles on mussels appear to achieve similar sizes (K. Suen personal observation; Appendix 2 Figure A2), suggesting that barnacles on mussels have lower net mortality than barnacles on bare rock, especially in the presence of *Mexacanthina*. Seasonality may also play a part in the observed feeding rates, as some studies have found whelks' activity fluctuates throughout the year (Rilov et al. 2005). It remains important to track any increase in the abundance of *Mexacanthina* in southern California so that impacts on native shellfish can be anticipated if average whelk densities reach peak observed values.

Despite historical emphasis on competitive interactions, our results reveal the importance of apparent facilitation between basal species. Competitive interactions between these two basal species have been extensively studied, including competition for space in the intertidal zone (Stephens and Bertness 1991); however, our findings also suggest a role for apparent facilitation between barnacles and mussels. On one side, the barnacle population that resided on mussels experienced less predation than barnacles on bare rock and thus benefited from the presence of the mussels. Likewise, mussels benefited from the presence of barnacles: because *Mexacanthina* chose to first consume the easy‐to‐access barnacles on bare rock, there was less predation pressure on nearby mussel populations.

Under the conditions tested, a range‐shifting species will preferentially prey on barnacles on bare rock rather than those on mussels, highlighting an indirect interaction between prey species. This finding demonstrates a mechanism through which the impacts of novel species might depend on the characteristics and composition of community members (Ricciardi et al. 2013; Kumschick et al. 2015), particularly when they include foundation species that can play multiple roles in the ecosystem. For invasive species, biotic mechanisms such as the novel species' trophic position and enemy/competitor release, and abiotic factors such as environmental heterogeneity and gradients can alter impacts (Ricciardi et al. 2013; Kumschick et al. 2015). Studies that look at species dispersing under climate change may benefit from also considering these mechanisms (Travis et al. 2013). However, context dependency studies on nonnative species have been found to require considerable resources and effort, suggesting that range shift‐focused studies would face similar issues (Kumschick et al. 2015). In order to identify in what cases management decisions would most benefit from information about the role of the recipient community and its environment, it would be ideal to use frameworks that identify common species traits (e.g., trophic level), impact mechanisms (e.g., consumption) and ecosystem characteristics that could lead to the most disruptive ecosystem changes (Blackburn et al. 2014; Gaertner et al. 2014). Impact assessment approaches are one such framework, as they involve compiling data on the impacts of novel species interacting in locations with diverse abiotic and biotic conditions (both in the species' historic and novel range) to predict community responses (Blackburn et al. 2014; Kumschick et al. 2015). Such impact assessments can therefore be adapted to create “watch lists” that identify species with the highest potential for negative impacts (Rockwell‐Postel et al. 2020; Wallingford et al. 2020). Studies of the role of community context can then be more targeted toward species for which impacts are most variable across locations, which will contribute to more accurate assessments for management responses (Kumschick et al. 2015). Such responses might include conservation efforts that support species (like mussels in the case of expanding whelks) that can help to mitigate range‐shift impacts (Giakoumi and Pey 2017; Pauchard et al. 2016; Ling and Johnson 2012).

## Author Contributions

**Kyle J. Suen:** conceptualization (equal), data curation (equal), formal analysis (lead), investigation (equal), methodology (equal), writing – original draft (lead), writing – review and editing (lead). **Ryan A. Beshai:** conceptualization (equal), data curation (equal), formal analysis (supporting), investigation (equal), methodology (equal), supervision (equal), writing – review and editing (equal). **Cascade J. B. Sorte:** conceptualization (equal), formal analysis (supporting), funding acquisition (lead), investigation (equal), methodology (equal), project administration (lead), resources (lead), supervision (equal), writing – review and editing (equal).

## Conflicts of Interest

The authors declare no conflicts of interest.

## Figures and Tables

**FIGURE A1:**
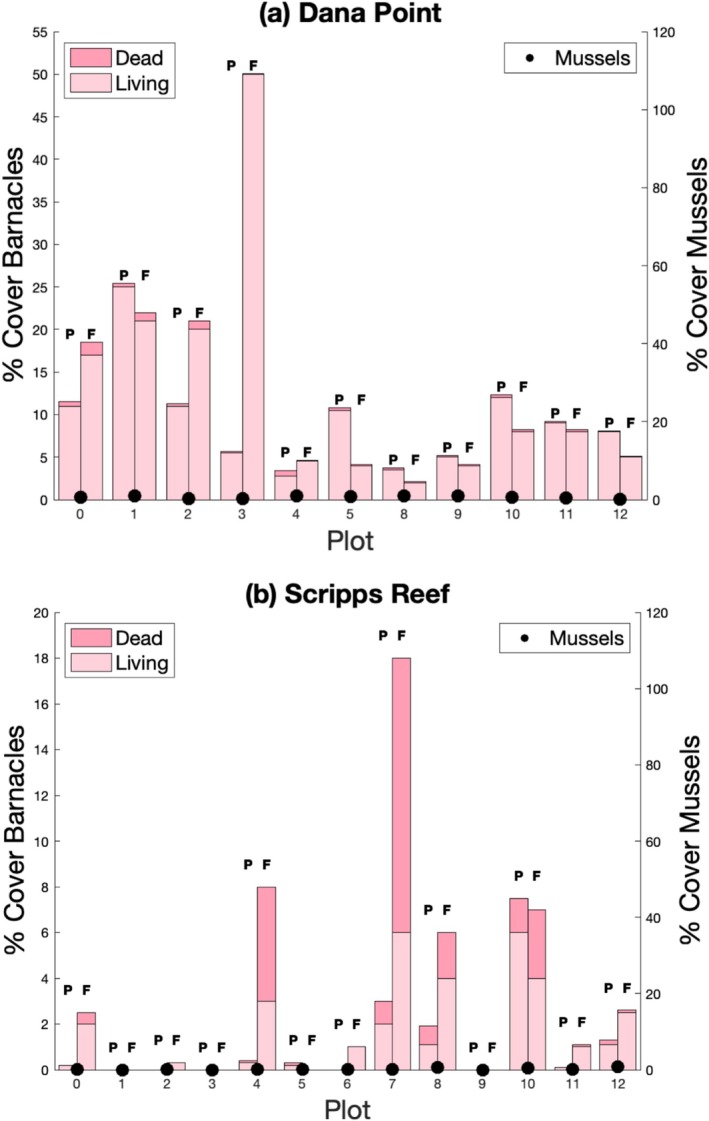
Left *y*‐axis: Percent cover of live (light pink) and dead (dark pink) barnacles on mussels within each plot at (a) Dana Point and at (b) Scripps Reef. Observations using photos are indicated by a “P”, and observations in the field are indicated by an “F”. Right *y*‐axis: Percent cover of mussels within each plot based on visual (photo) observations at (a) Dana Point and at (b) Scripps Reef (*n* = 12 points from 12 plots per site).

## Data Availability

The data that support the findings of this study are available at Dryad: https://doi.org/10.5061/dryad.69p8cz9ds.

## Appendix 1 Size Class Determination for *Mexacanthina*

**TABLE A1:** The total length of whelks in each size class in the caging experiment.

Site name	Small size (mm)	Medium size (mm)	Large size (mm)
Dana Point	< 29 mm	29–40 mm	> 40 mm
Scripps Reef	< 27 mm	27–35 mm	> 35 mm

*Note:* Size classifications were based on the size distribution of individuals encountered on the day of cage deployment.

## Appendix 2 Evaluating the Accuracy of Photographs for Barnacle Surveys

### Methods

Although the use of photos to record percent coverage of barnacles allowed for more precise measurements, the absence of the third dimension that is present in field observations made it difficult to account for barnacles hidden on the side of mussels. To determine if there was a difference between results from photos and field observations, subsequent surveys were performed at Dana Point (33.5497598, −117.7150156; on Sep. 18, 2024) and Scripps Reef (32.8715514, −117.2532418; on Sep. 22, 2024). At each site, a twelve‐meter transect was placed parallel to the coastline at the average height of the respective site's caging experiment and in an area where *Mexacanthina* could be found along the transect. A 0.1 m^2^ quadrat was placed at each whole meter mark of the transect, starting with the 0.0 m line. Within each quadrat, a photograph was taken for later image analysis and visual surveys were then conducted in the plot on‐site. There were a total of 24 observations (two sites × thirteen plots per transect, with the exception of plots at 6 and 7 m at Dana Point that were not surveyed due to unsafe tidal conditions).

Using visual estimates in the field and image analysis, the following metrics were quantified for each plot: (1) percent cover of live acorn barnacles on mussels and (2) percent cover of dead acorn barnacles on mussels. The difference between field and photo observations was assessed using *t*‐tests run in MATLAB (23.2.0.2485118 (R2023b) Update 6). Specifically, we compared values from field observations and photos for percent cover of live barnacles on mussels in each plot. We repeated this comparison for dead barnacles on mussels as well. We also compared the field‐observed proportion of mussels that are alive and on mussels derived from the field to the photo‐observed value. Finally, we evaluated the change in proportion of living barnacles on mussels as a function of mussel percent cover. The proportion of living barnacles was defined as the percent cover of living acorn barnacles on mussels divided by the total percent cover of barnacles on mussels (living + dead). All analyses were performed separately for each site, which we determined was appropriate given differences in habitat heterogeneity, prey species abundance, and prey dispersion patterns.

### Results

We found that there was not a significant difference between the two methods of observation, suggesting that the image analysis approach taken in our study was representative of patterns that we would have measured in the field (Table A2). When comparing the average percent cover of total barnacles living on mussels in a plot, the value found from visual observations in the field was not significantly different from the value found from image analysis (Dana Point *p* = 0.428; Scripps Reef *p* = 0.128; Figure A1). There was also no significant difference between the field‐derived value and the photo‐derived value of the average percentage of barnacles on mussels that were alive (as opposed to dead, empty tests), indicating that both observation methods yield similar results (Dana Point *p* = 0.5147; Scripps Reef *p* = 0.885; Table A2).

**TABLE A2:** Summary of observations made using both visual observations in the field and image analysis for Dana Point (*N* = 11 plots) and Scripps Reef (*N* = 13 plots).

Site	Dana Point		Scripps Reef	
Observation	Field	Photo	Field	Photo
Ave. % cover of barnacles on mussels	13.05 (±1.84)	9.39 (±1.84)	1.8 (±0.54)	0.85 (±0.46)
Ave. % of barnacles on mussels that were alive	96.70 (±0.64)	95.69 (±1.39)	50.90 (±11.45)	48.53 (±11.55)

*Note:* Values are average percentage ± standard error.
